# A novel genotype of avian hepatitis E virus identified in chickens and common pheasants (*Phasianus colchicus*), extending its host range

**DOI:** 10.1038/s41598-022-26103-3

**Published:** 2022-12-16

**Authors:** Miguel Matos, Ivana Bilic, Jana Tvarogová, Nicola Palmieri, Danuta Furmanek, Malwina Gotowiecka, Dieter Liebhart, Michael Hess

**Affiliations:** 1 Clinic for Poultry and Fish Medicine, Department for Farm Animals and Veterinary Public Health, University of Veterinary Medicine Vienna, Veterinaerplatz 1, 1210 Vienna, Austria; 2 MSD Animal Health, Warsaw, Poland

**Keywords:** Viral hepatitis, Viral pathogenesis, Viral epidemiology

## Abstract

In 2019, outbreaks of hepatitis-splenomegaly syndrome (HSS) were observed in six commercial layer chicken flocks, belonging to three different Polish farms, and characterized by increased mortality, hemorrhagic hepatitis with attached blood clots on the liver surface, and splenomegaly. Diseased flocks were initially investigated for the presence of avian hepatitis E virus (aHEV) – the etiological agent of HSS – by conventional reverse transcriptase polymerase chain reaction, which revealed aHEV sequences clustering separately from all known aHEV genotypes. Additionally, an aHEV genome was identified for the first time in common pheasants, from a flock in France, using Next Generation Sequencing. This genome clustered together with the Polish aHEVs here investigated. Complete genome aHEV sequences from the HSS outbreaks confirmed the divergent cluster, with a shared nucleotide sequence identity of 79.6–83.2% with other aHEVs, which we propose to comprise a novel aHEV genotype – genotype 7. Histology and immunohistochemistry investigations in the liver and spleen established an association between aHEV and the observed lesions in the affected birds, consolidating the knowledge on the pathogenesis of aHEV, which is still largely unknown. Thus, the present investigation extends the natural host range and genotypes of aHEV and strengthens knowledge on the pathogenesis of HSS.

## Introduction

The avian hepatitis E virus (aHEV) is the only member of the *Orthohepevirus B* species, within the genus *Orthohepevirus* in the family *Hepeviridae*^1^, and is the responsible agent for the hepatitis-splenomegaly syndrome (HSS) in chickens^2^. Initially referred to as big liver and spleen (BLS) disease in the first reported case from Australia^3^, HSS affects layer chickens and broiler breeders and is characterized by a drop in egg production up to 20%, which might be accompanied by an increase in weekly mortality of 1%^4^. During the *post-mortem* examination, 20% of the birds present severe hepato- and splenomegaly, and friable and mottled livers that may have subcapsular hematomas and attached blood clots on the surface^2^. However, the majority of aHEV infections result in a subclinical condition, in which some birds experience no clinical signs^5^.

Avian HEV has been reported in many countries worldwide, with seroprevalence studies in the USA, Spain, and Nigeria revealing 71–90% of chicken flocks and 15–80% of chickens positive for anti-aHEV antibodies^6–9^. Additionally, aHEV infection has been reported in wild birds^10,11^ and can be experimentally transmitted to turkeys^12^. Due to limitations in growing and isolating aHEV in vitro, the detection of aHEV is based on viral RNA detection by conventional reverse transcriptase polymerase chain reaction (RT-PCR), and antibodies by ELISA^2^.

The aHEV has a single-stranded, positive sense, RNA genome, with approximately 6.6 kb, consisting of three open reading frames (ORFs), and non-coding regions of 24 and about 130 nucleotides at the 5’- and 3’-end, excluding a poly(A) tail, respectively^13^. ORF 3, which codes for a small immunoreactive cytoskeleton-related protein^1^, is relatively conserved and, thus, ORF 1 and ORF 2 are used to investigate the genetic identity among different strains^13^. Phylogenetic analyses of genomic sequences have formerly revealed the existence of four distinct aHEV genotypes linked to geographic origin: genotype 1 includes aHEVs isolated from chickens in Australia, genotype 2 from the USA, genotype 3 from Europe and China, and genotype 4 from Taiwan and Hungary^4,5^. More recently, however, additional aHEV genotypes have been reported from China^14,15^. The lack of an efficient sytem for isolation and multiplication of aHEVs has resulted in a poor understanding of its replication and pathogenesis. Thus, epidemiological studies with a focus on genotyping and host tropism are important to fulfill such gaps in knowledge.

In the present study, we report nearly complete genome sequences and phylogenetic analysis of aHEVs belonging to a novel genotype, which were detected in various outbreaks of HSS in layer chicken flocks in Poland, but also in pheasants from France.

## Methods

### Outbreak description and field investigations

In 2019, several commercial layer chicken flocks in Poland, belonging to three different multi-age poultry farms—A, B, and C (Table 1)—experienced increased mortality. During *post-mortem* investigations, samples of liver, spleen, and bile of diseased chickens were collected in FTA® cards and shipped for further laboratory investigations. In three flocks, cloacal swabs were also collected from affected birds (Table 1).

**Table 1:** List of samples from Polish chicken flocks suffering from HS syndrome that were investigated for aHEV RNA by conventional RT-PCR, and corresponding results.

Sample ID	Flock^a^	Age at sampling (weeks)	Sample			Conventional RT-PCR	
			Material	No.^b^		Helicase gene	Capsid gene
19-13931	A1	45	FTA card (liver)	1	(4)	+ ^c^	−^d^
			FTA card (spleen)	1	(4)	+	−
			FTA card (bile)	1	(4)	+	+
19-13932	A2	32	FTA card (liver)	1	(4)	−	−
			FTA card (spleen)	1	(4)	−	−
			FTA card (bile)	1	(4)	+	−
19-13933	A3	43	FTA card (liver)	1	(4)	+	−
			FTA card (spleen)	1	(4)	+	+
			FTA card (bile)	1	(4)	+	−
19-27329	B1	37	FTA card (liver)	1	(3)	+	−
19-27330			FTA card (spleen)	1	(3)	+	−
19-27331			FTA card (bile)	1	(3)	+	+
19-27332			FTA card (cloaca)	1	(4)	+	+
				2	(4)	+	−
				3	(4)	+	−
				4	(4)	+	+
				5	(4)	+	−
19-27335	B2	68	FTA card (liver)	1	(2)	+	−
19-27336			FTA card (spleen)	1	(2)	+	−
19-27337			FTA card (bile)	1	(2)	+	+
19-27338			FTA card (cloaca)	1	(4)	−	−
				2	(4)	+	−
				3	(4)	+	−
				4	(4)	+	−
				5	(4)	+	−
19-27341	C	27	FTA card (liver)	1	(4)	−	−
				2	(4)	−	−
19-27342			FTA card (spleen)	1	(4)	+	−
				2	(4)	+	−
19-27343			FTA card (bile)	1	(4)	+	−
				2	(4)	+	−
19-27344			FTA card (cloaca)	1	(4)	+	−
				2	(4)	−	−
				3	(4)	+	−
				4	(4)	+	−
				5	(4)	+	−

^a^Flocks with the same letter belong to the same farm.

^b^Sample number (number of corresponding sampled birds).

^c^Positive.

^d^Negative.

In a distinct case, commercial pheasant flocks from France suffered from outbreaks of hepatitis with high mortality. The description of the outbreaks and performed investigations, including collected samples and subsequent analyses, have been recently reported elsewhere^16^.

### Histopathology

Liver and spleen samples were processed for histology, being initially fixed in a 4% neutral buffered formaldehyde solution (SAV LP GmbH, Flintsbach, Germany), followed by a dehydration procedure and paraffin embedding. The formalin-fixed paraffin-embedded (FFPE) samples were then cut into sections of 4 µm with a microtome (Microm HM 360; Microm Laborgerate GmbH, Walldorf, Germany), mounted on glass slides, and stained with hematoxylin and eosin (H&E) for microscopic assessment.

### Immunohistochemistry for the detection of aHEV

A truncated recombinant aHEV capsid protein, designated ORF2-1, was expressed as previously reported^17^ and inoculated in rabbits to produce polyclonal antibodies. For immunohistochemistry (IHC) detection of aHEV in tissues, additional sections of FFPE liver and spleen samples were obtained (4 µm) by a microtome (Microm HM 360) and mounted on positively charged glass slides (Superfrost plus; Menzel-Gläser, Braunschweig, Germany). Liver and spleen samples of specific-pathogen-free chickens, both healthy birds and those suffering from hepatitis (due to fowl adenovirus infection), were included to study the specificity of the polyclonal serum. After the first step of dewaxing and rehydration, the slides were heated in citrate buffer (pH 6.0), for antigen retrieval. The activity of endogenous peroxidase was then blocked with 1.5% H_2_O_2_ in methanol, for 30 min. As a blocking step, the sections were incubated with a 1:10 dilution of normal goat serum (Vector Laboratories, Burlingame, USA) mixed with 2% bovine serum albumin (Roche Diagnostics GmbH, Mannheim, Germany) for 60 min, at room temperature, in a humidified chamber. Next, the slides were incubated overnight, at 4 °C, with the primary antibody (purified rabbit polyclonal anti-ORF2 aHEV serum) at three dilutions: 1:500, 1:1000, and 1:1500. As a control for the primary antibody, additional sections were incubated with PBS. The sections were then extensively washed in PBS, and incubated with a 1:400 dilution of biotinylated anti-rabbit IgG (Vector Laboratories) for 30 min, followed by Vectastain ABC Kit (Vector Laboratories) for 60 min. The reaction was visualized with a DAB Substrate Kit for peroxidase (Vector Laboratories). After counterstaining with Mayer’s Hematoxylin (Merck, Darmstadt, Germany) and dehydration, the sections were mounted under coverslips with Neomount (VWR, Vienna, Austria).

### RNA extraction and RT-PCR

RNA was extracted from FTA® card pooled samples of liver, spleen, bile, and cloacal swab samples (Table 1) using the IndiSpin Pathogen Kit (Indical Bioscience, Leipzig, Germany), according to the manufacturer’s instructions. Extracted total RNA was eluted in 50 µl ultra-purified water or the elution buffer included in the kit. The samples were then investigated by RT-PCR using the OneStep RT-PCR kit (Qiagen, Vienna, Austria), according to the manufacturer’s instructions. Primer pairs targeting the Helicase gene (Helicase F/R)^6^, within ORF 1, and the Capsid gene (Forw/Rev1_C-BLSV)^13^, within ORF 2, were used in a final concentration of 1 µM. Negative extraction and PCR controls were included in all RT-PCR reactions. The PCR products were analyzed by gel electrophoresis in a 1.5% (w/v) Tris acetate-EDTA-agarose gel at 100 V for 60 min, stained with GelRed® (Biotium, Vienna, Austria), and visualized under ultraviolet light (BioRad Universal Hood II; Bio-Rad Laboratories, Hercules, CA). Expected PCR products were excised and purified from the gel, with QIAquick Gel Extraction Kit (Qiagen), according to the manufacturer’s instructions, and sequenced directly by the Sanger method using PCR primers (LGC Genomics, Berlin Germany).

### Next generation sequencing

Sample 19-03914, which consisted of FFPE liver and bursa of Fabricius, derived from a pheasant flock in France, suffering from hepatitis and high mortality, was investigated by deep sequencing using the Illumina NextSeq platform, as it was comprehensively described elsewhere^16^. To extract only contigs with aHEV specific sequences, all viral contigs with size ≥ 100 bp were compared against a database set up to contain all available *Orthohepevirus B* sequences, using the BLASTn algorithm. Based on the best BLASTn-scores (E-value ≤ 10^–10^), metagenomics contigs of up to near-complete genome length of an aHEV strain were identified.

### Obtaining near-complete genome sequences

Based on the aHEV sequence identified in the pheasant sample, eight primer pairs covering the nearly complete genome were designed and applied to chicken samples (Table 2). All PCRs to obtain the whole aHEV genomes of samples 19-13931 and 19-27337 were performed with the OneStep RT-PCR kit (Qiagen) and 15 pmol of each primer (Table 2). The thermal profile was as follows: 30 min at 50 °C for reverse transcription, 15 min at 95 °C for initial PCR activation and denaturation, followed by 40 cycles of 30 s at 94 °C, annealing temperature as in Table 2 for 30 s, and 72 °C for 90 s. Final elongation took place at 72 °C for 10 min. All PCR products were analyzed by gel electrophoresis in a 1% (w/v) Tris acetate-EDTA-agarose gel at 100 V for 60 min, stained with GelRed® (Biotium), and visualized under ultraviolet light (BioRad Universal Hood II; Bio-Rad Laboratories). Expected PCR products (Table 2) were excised and purified from the gel with the QIAquick Gel Extraction Kit (Qiagen), according to the manufacturer’s instructions. Purified fragments were cloned into the pCR®4-TOPO® vector by using the TOPO TA Cloning Kit for sequencing (Invitrogen, Thermo Fisher Scientific, Vienna, Austria) according to the manufacturer’s instructions. At least three clones per cloning reaction sample were sequenced by Sanger sequencing using M13 primers (LGC Genomics). The consensus sequence derived from three clones was used for the assembly of near-complete genomes by the Accelrys Gene software, version 2.5 (Accelrys, San Diego, CA).

**Table 2:** List of primer pairs used to obtain the complete genome sequences of samples 19-13931-bile and 19-27337-bile, with respective product length and position regarding original sample 19-03914, and annealing temperature.

Primer pair	Forward (5′ → 3′)	Reverse (5′ → 3′)	Product length (bp)	Genome positions (sample 19-03914)	Ta^a^ (°C)
Ph_aHEV-pp1	GAGTCCAAGGGGGTTAAAAC	ACAAAATGGCAGCCTATnGC	767	35–801	57
Ph_aHEV-pp2	TACCTTACCCAGCAGCAGAC	GGCAAGCTGACGATAGAACC	1182	122–1303	58
Ph_aHEV-pp3	CCGCGTATCTCACAGTyTG	CCGGCACTAATTAAAGGGC	1069	1131–2199	59
Ph_aHEV-pp4	TCCTTGCTGTGCTACCrTC	GGGATTTGACTGTTTAGCCAC	1082	1866–2947	58
Ph_aHEV-pp5	ACTATCCGGCCATTACCACvAC	AACCAGGGGCCAAAAAGnGC	952	2838–3789	58
Ph_aHEV-pp6	CGGATTGTTCCCGCATwAC	CAGCACATAGCAAGCATGAG	1086	3654–4739	57
Ph_aHEV-pp7	CGAAACTCTCACGACTAAyAG	CCATTGGCATTCCCAACAG	1193	4558–5750	58
Ph_aHEV-pp8	ATTTGTCACCCGGCAAyAC	ACCCCAAAACCACATTATyAAG	1075	5462–6536	57
Ph_aHEV-pp9	CTGGCAGCACCATGATGG	GACTCGAGTCGACATCGAT_16_ (AP-Primer)	600	5959-end	55

^a^Annealing temperature.

### Phylogenetic analysis

All near-complete and complete genomes representing the known genotypes of aHEV were retrieved from GenBank (19th, July 2022) to confirm the phylogenetic relationship of the newly discovered aHEV genome in samples 19-03914, 19/13931, and 19/27337. Available and newly obtained aHEV genome sequences were aligned with the MAFFT method as implemented in the MegAlig Pro module of Lasergene v17.3 software (DNASTAR, Madison, WI, USA) followed by a selection of conserved blocks using the online tool GBlocks https://ngphylogeny.fr/tools/tool/276/form)^18^. Phylogenetic analysis was performed using the Maximum Likelihood (RAxML) approach as implemented in the MegAlign Pro module of the Lasergene v17.3 software (DNASTAR). The robustness of the phylogenetic tree was analyzed by bootstrap re-sampling using 500 replicates. Bootstrap values ≥ 75% were considered significant. Preliminary phylogenetic analyses with partial sequences from helicase and capsid genes of samples 19-03914, 19-13931, 19-13933, 19-27331, and 19-27337, were performed by the Neighbor-Joining (BIONJ) approach using the MegAlign Pro module of the Lasergene v17.3 software (DNASTAR). Sequence distances were derived by Uncorrected Pairwise distance metric and global gap removal as implemented in the MegAlign Pro module of Lasergene v17.3 software (DNASTAR).

## Results

### Clinical features of the HSS outbreaks

**Figure 1:**
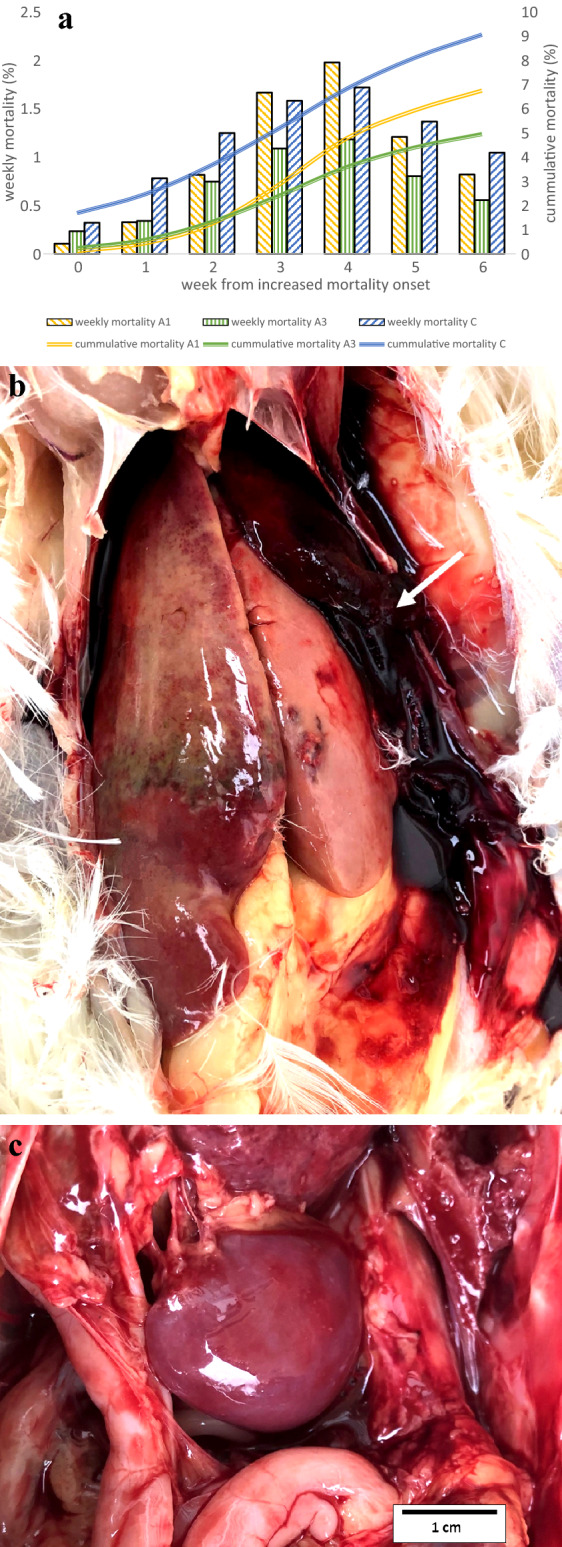
Characterization of aHEV field outbreaks in Poland. Weekly and cumulative mortality values (%) in three affected flocks—A1, A3, and C (**a**), and predominant *post-mortem* findings in the liver (**b**), where hemorrhagic hepatitis with hepatomegaly and clotted blood ( →) can be observed, together with splenomegaly (**c**).

Mortality in selected flocks A1, A3, and C reached the highest weekly values of 2%, 1.2%, and 1.7%, four weeks after the onset of increased mortality, with cumulative values of 6.7%, 5%, and 9%, respectively, being recorded after six weeks (Fig. 1a). The affected flocks were between 27 and 68 weeks of age and were kept in a cage system. *Post-mortem* investigations revealed friable, swollen livers, with hepatitis, subcapsular hematomas, and /or attached blood clots on the surface, and splenomegaly (Fig. 1b, c).

### Histopathology analysis

**Figure 2:**
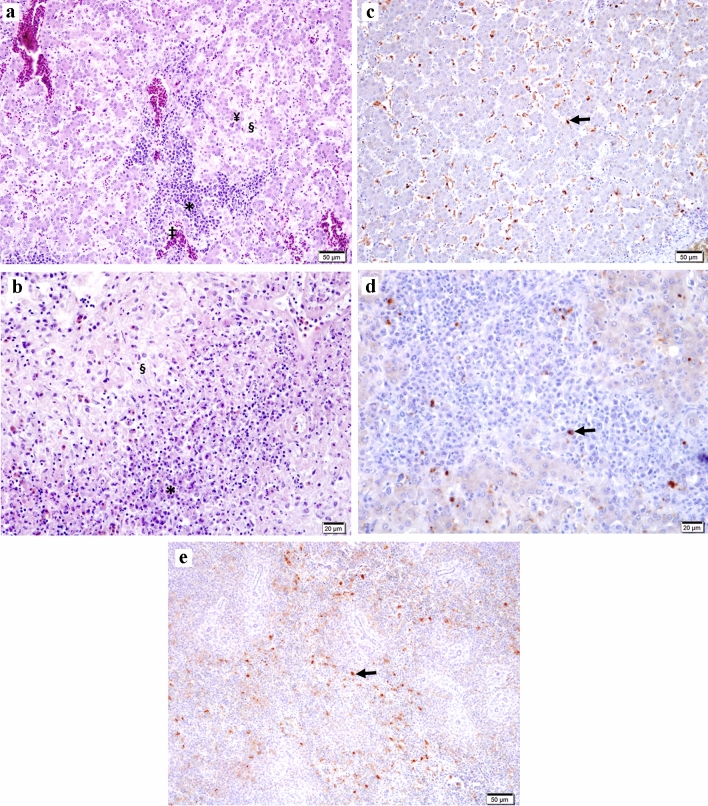
Histopathological and IHC investigations on samples from chickens suffering from HSS. In liver (**a**), infiltrations of heterophils and mononuclear cells can be observed (*), together with compression and atrophy of the hepatic cords and hepatocytes (¥) due to the accumulation of proteinaceous material in the sinusoids indicating amyloid (§). Sinusoidal congestion (‡) can also be noticed (H & E; bar = 50 µm). The spleen (**b**) presents lymphocytic infiltration (*), with extensive disruption of the splenic tissue by homogenous, eosinophilic material most suggestive of amyloid protein (§) (H & E; bar = 20 µm). Positive signals for the aHEV ORF2 ( →) are shown by IHC in the sinusoids, in the liver (**c**; bar = 50 µm), and in association with areas of mononuclear cell infiltrations (**d**; bar = 20 µm), as well as in the peri-ellipsoidal zones throughout the spleen (**e**; bar = 50 µm).

The histology assessment of liver samples revealed multifocal areas of coagulative necrosis or generalized necrosis, infiltrations of heterophils and mononuclear cells, and several areas of hemorrhages. Additionally, sinusoidal deposits of eosinophilic, amorphous, hyaline material, suggestive of amyloid, were observed, which disrupted the hepatic plates (Fig. 2a). Some liver samples also presented granulomas and colonies of rod-shaped bacteria. Likewise, the analysis of spleen samples revealed multifocal to generalized areas of necrosis, infiltration of heterophils, and hemorrhages. In addition, severe and extensive disruption of the splenic tissue by homogenous, amorphous, eosinophilic, proteinaceous material consistent with amyloid, was a common finding (Fig. 2b).

Positive signals for aHEV ORF2 were detected by IHC in both liver and spleen samples of affected chickens. In the liver, positive signals were observed in the hepatic sinusoids and in accumulations of mononuclear cells (Fig. 2c, d), while in the spleen positive signals were more present in the peri-ellipsoidal zones, throughout the organ (Fig. 2e). The positive signals were more distinct and with the least background with a primary antibody dilution of 1:1000. No positive signals were observed in the controls investigated.

### RT-PCR investigations

Samples of liver, spleen, bile, and cloaca were investigated for aHEV RNA by RT-PCRs, targeting the helicase and capsid genes. Out of 36 samples investigated, 30 were positive for the aHEV helicase gene—4 out of 7 (4/7) liver samples, 6/7 spleen samples, 7/7 bile samples, and 13/15 cloacal swab samples—and six were positive for the aHEV capsid gene—3/7 bile samples, 2/15 cloacal samples and 1/7 spleen sample (Table 1).

### Discovery of a novel aHEV genotype

**Figure 3:**
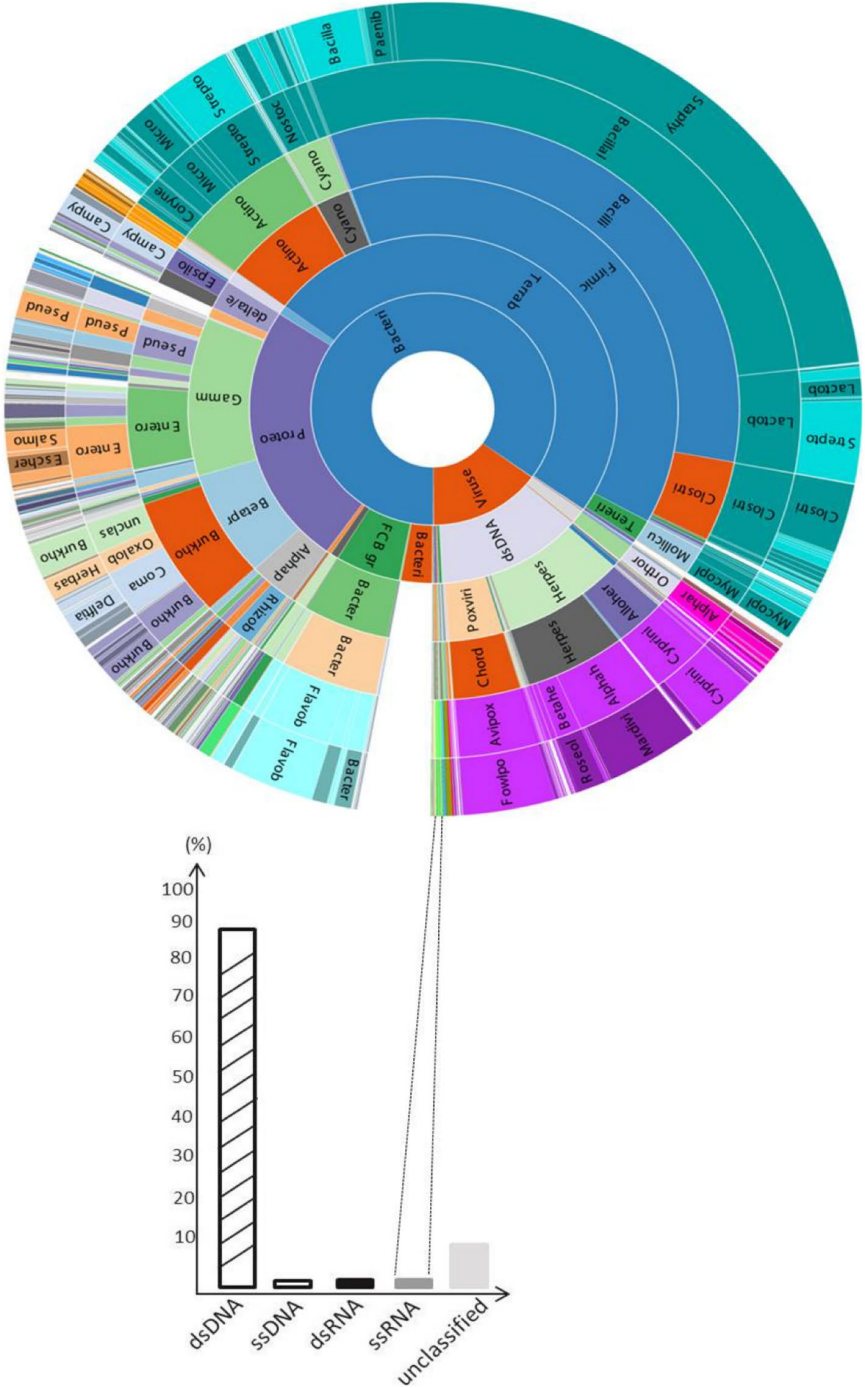
Metagenomics profiling of the 19-03914 liver sample from a pheasant flock in France. The metagenomics profiling represents all families in different color and/or different color gradients. All identified de novo assembled virus contigs from metagenomics profiling are shown in the bar graph, where the percentage of the coverage of double-stranded (ds), single-stranded (ss) viral DNA and RNA, as well as unclassified viruses is shown.

Initial de novo metagenome assembly of sample 19–03914 from pheasants yielded 67,302,080 reads, which were assembled into 3881 contigs of lengths varying from 362 to 223,074 nucleotides. The obtained metagenomics profiling identified different families of bacteria and viruses. Furthermore, the viral contigs were classified into five different categories with the largest portion (86%) corresponding to double-stranded (ds) DNA viruses (Fig. 3). Among the others, a genome of aHEV was identified, and the presence of aHEV RNA in the sample 19-03914 was, then, confirmed by RT-qPCR, according to a previously published protocol^19^. The assembled sequence of 6639 nucleotides in length covered the near-complete aHEV genome, excluding only the terminal portions of the 5' and 3' non-coding regions (NCR). The complete genome sequence was submitted to the NCBI database under the accession number ON922634.

Phylogenetic analyses using partial sequences of helicase and capsid genes derived from RT-PCR products revealed that aHEV sequences from chicken samples 19-13931, 19-13933, 19-27331, and 19-27337, cluster together with the 19-03914 pheasant aHEV sequence (Supplementary Fig. S1a, b). Therefore, based on the 19-03914 aHEV sequence, a set of primers amplifying overlapping 1 kb regions of the nearly complete aHEV genome was designed and applied on samples 19-13931 and 19-27337 (Table 2). Obtained fragments were sequenced by the Sanger method, and the assembled nearly complete genome sequences of 6460 bp were submitted to the NCBI database under the accession numbers ON922632 and ON922633, respectively.

### Genome structure and phylogenetic analysis

Overall, the genomic organization of the 19-03914, 19-13931, and 19-27337 strains corresponded to those of known aHEVs. Flanked by the partial 5` and noncoding regions, complete open reading frames ORF1, ORF2, and ORF3 were identified in their respective order in the genome, with the ORF1-translated polyprotein enclosing most amino acid substitutions among all ORF-translated proteins due to the hypervariable polyproline region (Supplementary Fig. S2).

**Figure 4:**
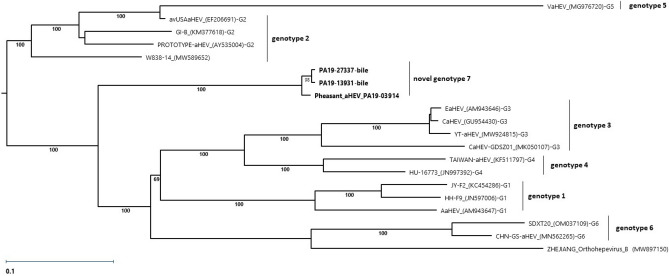
Phylogenetic analysis of *Orthohepevirus B* genomes. The analysis was performed using the Maximum Likelihood method (RAxML) and was based on the alignments of conserved sites that included 6405 positions in the final dataset. The unrooted tree with the highest log likelihood (− 43,903.392991) is shown. This analysis involved 20 nucleic acid sequences. All genomes from the Genbank are labelled with the name, accession number in parenthesis, and the designation of the corresponding genotype as G-number. Sequences obtained in the present study are labelled in bold. The robustness of the tree was tested by the bootstrap analysis with 500 iterations and the percentage of trees in which associated taxa clustered together is shown next to the branches. Evolutionary analysis was conducted in the MegAlign Pro module of Lasergene v17.3 software (DNASTAR, Madison, WI, USA).

The phylogenetic analysis was based on whole genome sequences belonging to the species *Orthohepevirus B*. All aHEV genomes reported here clustered together in a separate branch, representing a new putative aHEV genotype (Fig. 4), with a shared nucleotide sequence identity of 98–99.3%, and a 79.7–83.3% nucleotide identity with other aHEVs (Supplementary Table S1—Percent identity complete genomes).

## Discussion

Since its first clinical appearance in 1988 in Australia, serological and molecular studies have revealed a worldwide prevalence of aHEVs in chicken flocks, which however does not translate necessarily in a clinical condition^5^. Additionally, histopathological lesions produced by aHEV are etiologically unspecific^2^. The impressive liver lesions noticed macroscopically can easily be confounded with amyloidosis, as described in context with the application of bacterial oil-emulsion vaccines^20,21^. Therefore, in the present study, we established an IHC protocol, with a primary polyclonal rabbit anti-aHEV ORF2 antibody, to confirm the presence of aHEV antigen in organs presenting pathological changes. Our investigations revealed positive signals in RT-PCR-positive samples of liver and spleen from chickens suffering from HSS, establishing an association between aHEV and the observed lesions. The distribution of the positive signals in these organs reinforced the findings of previous studies^22,23^, with aHEV antigen being detected mainly in the hepatic sinusoids in the liver, while in the spleen aHEV antigen was not confined to a particular area. Such findings are important to consolidate the knowledge on the pathogenesis of aHEV, which is still largely unknown. It is established that viral replication initially takes place in gastrointestinal tissues before reaching the liver^5^, and our results seem to further indicate that sinusoidal cells in the liver might play a significant role in the aHEV replication. Additionally, the scattered pattern of antigen observed in the spleen, leading to splenomegaly, suggests the involvement of mononuclear cells in the replication of aHEV.

Since the knowledge on aHEV pathogenesis and aHEV strains’ pathogenicity is limited by the lack of an appropriate lab propagation system^5^, it is important to monitor field aHEVs circulating in the bird populations and record associated outbreaks. More recently, a novel genotype identified in China was associated with a more severe form of the disease in chickens denominated by hepatic rupture hemorrhage syndrome (HRHS), leading to a cumulative mortality of 15% in the affected flocks^24^. Similarly, the HSS outbreaks reported in the present study lead to high mortality rates of up to 2% per week, and up to 9% cumulatively. More detailed epidemiological investigations would be needed, including clinically healthy birds, to correlate between the genotype and pathogenicity of a certain aHEV strain.

After being identified as the causative agent of the BLS disease/HSS in chickens^25,26^, genetic characterization studies revealed, throughout the years, heterogeneity of aHEV field strains^6,13,27,28^. In this regard, four distinct aHEV genotypes were proposed initially, which at that time correlated to the different geographical locations where the virus was identified^4^. More recently, two additional genotypes have been proposed from the analysis of the complete genome of aHEV strains identified in China^14,15^. In the present investigation, we report nearly complete aHEV genomes identified in pheasants and layer chickens, which belong to a novel aHEV genotype—genotype 7.

Initially, we identified the new aHEV genome by NGS in organ samples of pheasants from France suffering from hepatitis, caused by a novel *Chaphamaparvovirus*^16^. Since the presence of aHEV RNA was only circumscribed to one pheasant-rearing farm out of 15 investigated, the finding was considered accidental and negligible to the clinical outcome as it was undoubtedly attributed to the parvovirus. Additionally, it is well known that birds can be PCR-positive for aHEV in the absence of associated disease^5^. Nevertheless, this is the first time that aHEV is reported in common pheasants (*Phasianus colchicus*), extending its natural host range. Such finding, however, is not unexpected as in the last 6 years different studies identified aHEVs in birds other than chickens, demonstrating a much broader natural host range for aHEV than previously considered^10,11,29,30^.

Differently from the findings in pheasants, six layer chicken flocks, with ages between 27 and 68 weeks, belonging to three different farms in Poland, presented a clinical picture compatible with classically described HSS, which included increased mortality, hemorrhagic hepatitis with attached blood clots on the liver surface, and splenomegaly. Diseased flocks were initially investigated for the presence of aHEV by conventional RT-PCR targeting helicase and capsid genes, with the former presenting higher positivity rates than the latter. This is likely because the viral RNA coding for ORF1 (helicase) and ORF2 (capsid) are replicated in different amounts, depending on the stage of infection, with high viral load samples being positive in both RT-PCRs^31^.

Preliminary phylogenetic analyses of partial helicase and capsid sequences obtained from RT-PCR products suggested that the aHEVs identified in the Polish chicken flocks, together with the 19-03914 aHEV genome from pheasants, compose a separate cluster within all known aHEV genotypes. Complete genome aHEV sequences from the Polish cases were obtained to confirm the divergent cluster, which we propose to comprise a novel aHEV genotype. The comparative analysis of all available genomes of *Orthohepevirus B* species identified genome identity values in a range from 77.7 to 100%, and a 15–20% variation in the complete nucleotide sequences between aHEV genotypes, criteria that the aHEV complete nucleotide sequences here reported fulfil.

Previously, aHEV genotypes 2, 3, and 4 were identified in birds in Poland, revealing that aHEV is widely spread in Polish chicken flocks^11,31–33^. Interestingly, some aHEV partial helicase sequences recently reported from broilers and laying hens in Poland, which were assigned to genotype 4^11^, seem to be phylogenetically related to the aHEV genomes reported in the present study. Applying the BLAST search algorithm with newly identified genomes, recognized the above-mentioned genotype 4 isolates as sequences with high nucleotide identity (98.2–99.7%). This would imply that the novel aHEV genotype reported here is prevalent in Polish flocks; however, to verify such hypothesis analyses using complete genomes from such cases would be necessary.

In conclusion, here we report nearly complete genomes belonging to a novel aHEV genotype, which were identified in chickens, but also for the first time in pheasants, extending the natural host range of aHEVs. In chickens, the outbreaks were characterized by high mortality, and anatomic-pathological lesions compatible with HSS, and the association of aHEV in the clinical picture was confirmed by IHC in the liver and spleen of affected birds. Hence, the present report extends the host range and genotypes of aHEV, consolidating knowledge on the pathogenesis of HSS, and contemplates important questions on aHEV pathogenesis that shall be considered in future studies.

## Supplementary Information

Supplementary Figures.

Supplementary Table S1.

## Data Availability

The dataset generated and analyzed during the current study is available in the GenBank repository. The sequences are deposited under the following accession numbers: ON922632, ON922633, and ON922634. Additional data that support the findings of this study are available from the corresponding author upon reasonable request.
