# Supplementary material for: A novel genotype of avian hepatitis E virus identified in chickens and common pheasants (Phasianus colchicus), extending its host range
Source: Sci Rep. 2022 Dec 16;12:21743. doi: 10.1038/s41598-022-26103-3 (PMC9758205; doi:10.1038/s41598-022-26103-3)
Supplement: Supplementary file 1 — Supplementary Figures. [file 41598_2022_26103_MOESM1_ESM.pdf]

Supplementary Figure S1a

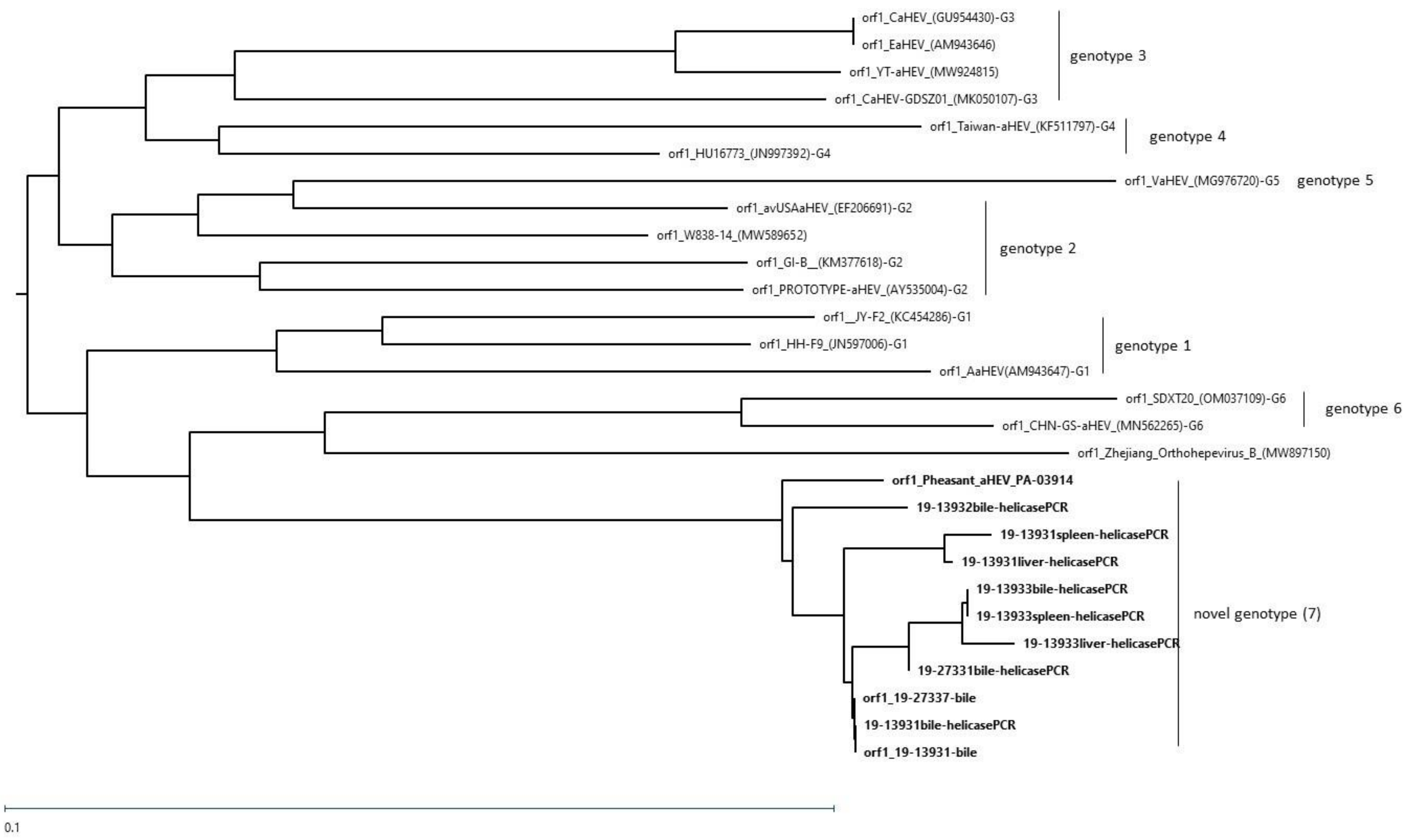

Supplementary Figure S1b

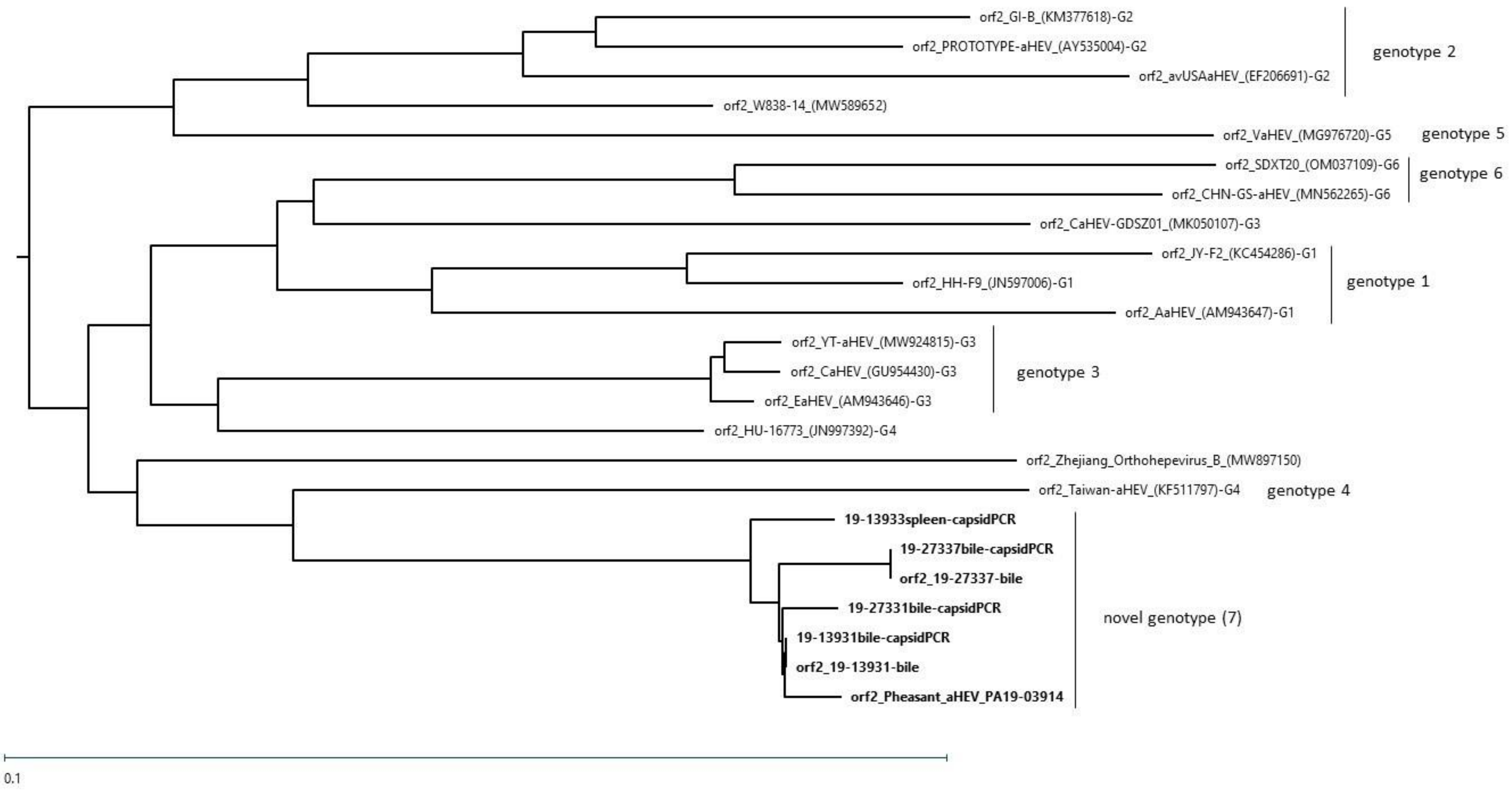

Supplementary Figure S1. Phylogenetic analysis of Polish flocks' samples with representative strains of all *Orthohepevirus B* genotypes including Pheasant-aHEV. a) Analysis based on a partial helicase sequence that included 145 positions in the final dataset and involved 28 nucleic acid sequences. b) Analysis based on a partial capsid sequence that included 166 positions in the final dataset and involved 24 nucleic acid sequences. Both analyses were performed using the Neighbor-Joining (BIONJ) method with Uncorrected Pairwise distance metric and global gap removal and were conducted in the MegAlign Pro module of Lasergene v17.3 software (DNASTAR, Madison, WI, USA). Sequences generated within the present study are highlighted in bold. All other sequences were derived from complete genomes deposited in the Genbank and are labeled with the name, accession number in parenthesis, and the designation of the corresponding genotype as G-number.

Supplementary Figure S2a

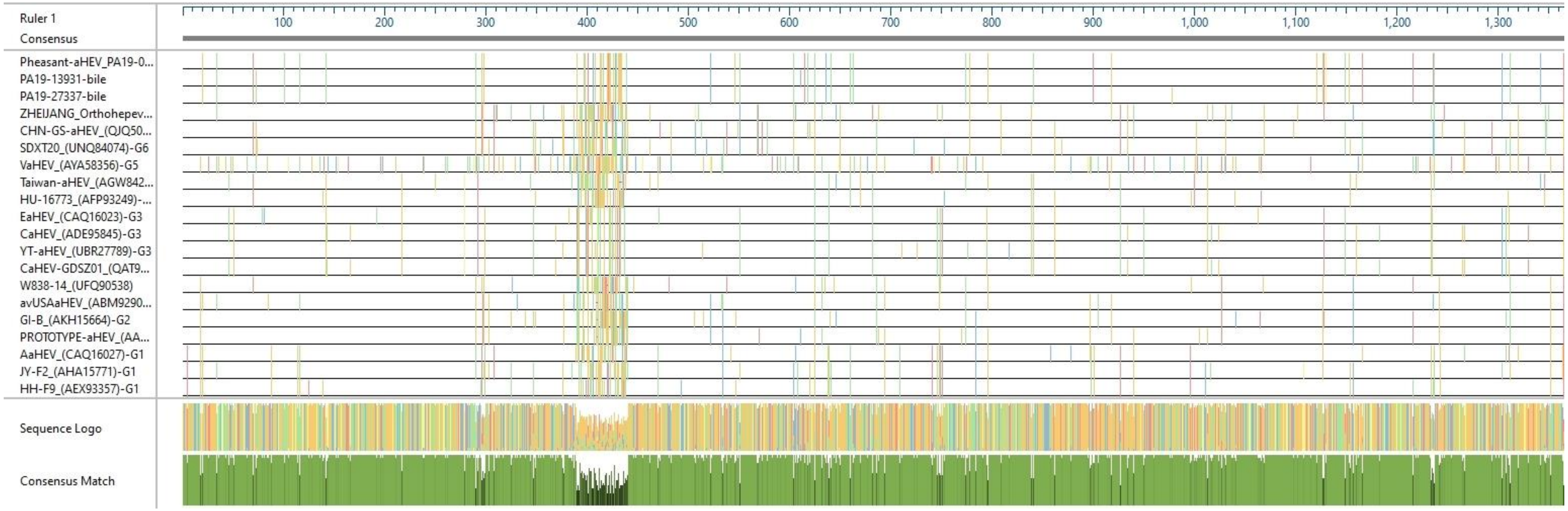

Supplementary Figure S2b

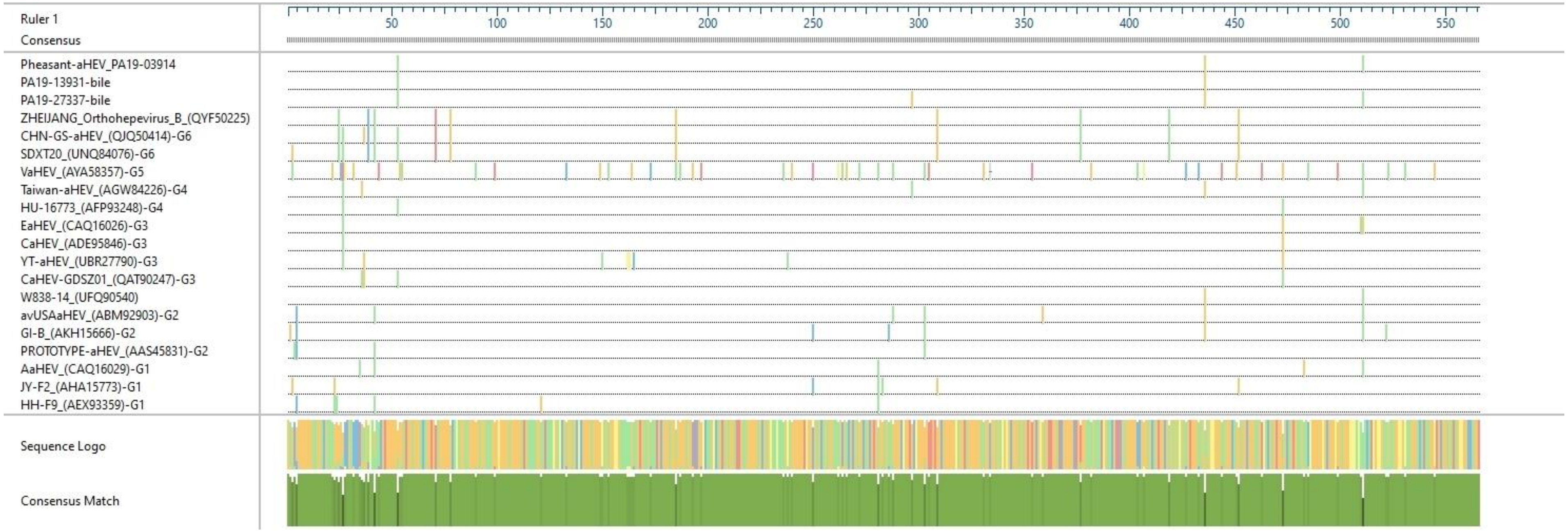

Supplementary Figure S2c

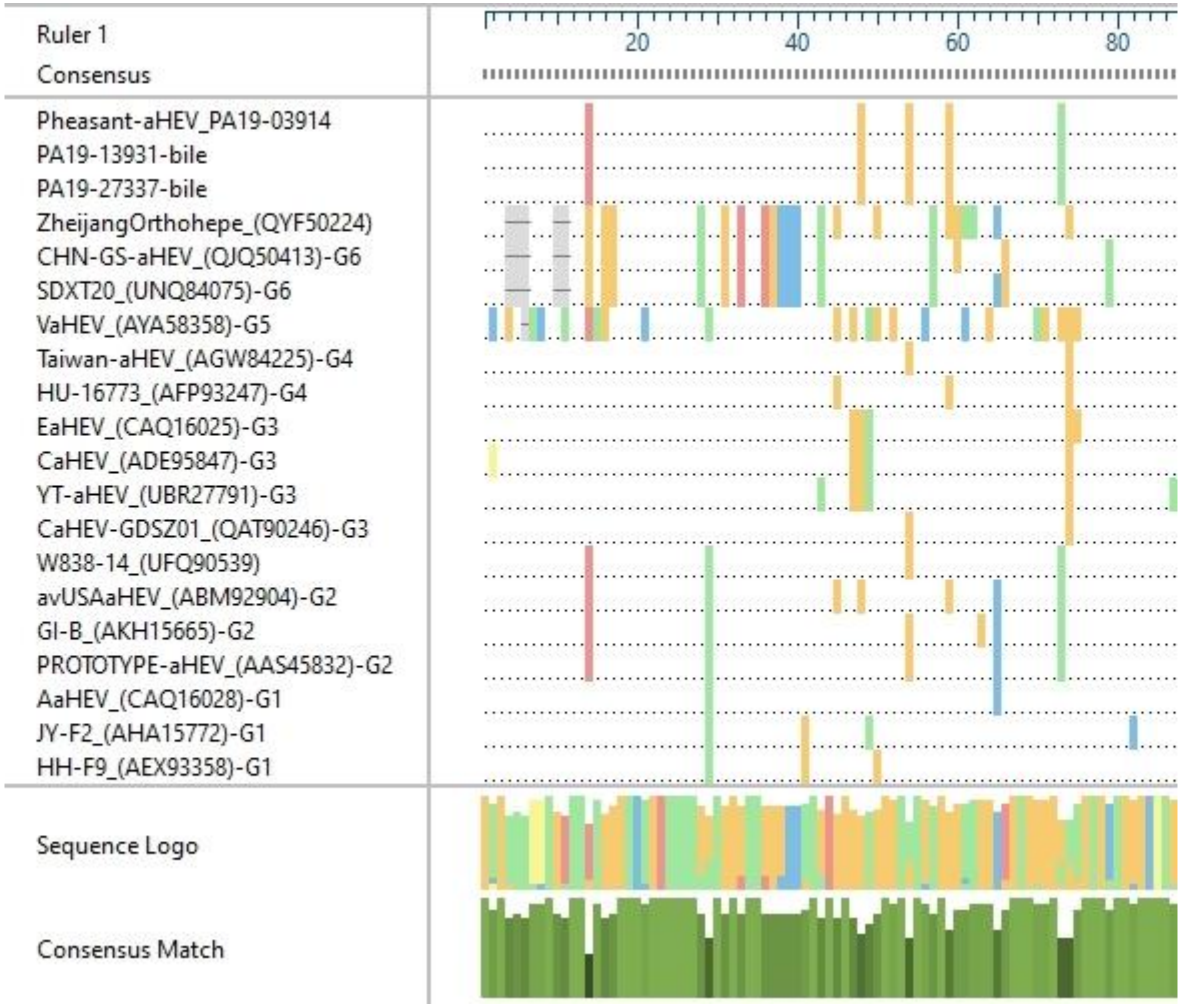

Supplementary Figure S2. Amino acid alignment of a) ORF1-polyprotein, b) ORF2-capsid protein and c) ORF3-cytoskeleton-related protein. Amino acid alignments were done in the MegAlign Pro module of Lasergene v17.3 software (DNASTAR, Madison, WI, USA). Positions that display differences to the consensus sequence are shown as vertical bars, conserved positions are shown as dots, and gaps as vertical lines. Tracks below the alignment display amino acid composition colored by chemistry and consensus match measurement (%).
